# Association between Y-chromosome AZFc region micro-deletions with recurrent miscarriage

**Published:** 2013-05

**Authors:** Saeede Soleimanian, Seyyed Mahdi Kalantar, Mohamad Hasan Sheikhha, Mohamad Ali Zaimy, Azam Rasti, Hossein Fazli

**Affiliations:** 1 *Molecular Medicine Research Center, Hormozgan Univercity of Medical Sciences, Bandarabbas, Iran.*; 2 *Research and Clinical Center for Infertility, Shahid Sadoughi University of Medical Sciences, Yazd, Iran.*; 3 *Department of Microbiology, Hamedan University of Medical Sciences, Hamedan, Iran.*

**Keywords:** *Recurrent abortion*, *Y-chromosome micro deletions*, *Multiplex PCR*

## Abstract

**Background: **In human, about 25% of implanted embryos are losing 1-2 week following attachment to the uterus. A subset of this population will have three or more consecutive miscarriages which define as repeated pregnancy loss (RPL). Introducing the assisted reproductive technologies (ARTS) made a chance for infertile couples to solve their childless problem.

**Objective:** This study was conducted to evaluate the incidence of Y-chromosome AZF region's micro-deletions in male partners of couples with recurrent miscarriage (RM).

**Materials and Methods: **Thirty male partner of couples with RM and thirty infertile males, who referred to the Yazd Research and Clinical Center for Infertility were recruited to this study. In addition, 30 healthy men were screened as a control group from the same center. After DNA extraction using salting out method, the multiplex-PCR was done for amplifying 8 known STSs proximal to the AZF region of the Y-chromosome. The results were compared between the groups using Fisher's exact t-test and p<0.05 was considered statistically significant.

**Results:** Of the 30 infertile males, 5 (16.6%) cases were associated with the AZF region micro-deletions of DYF87S, DYF84S1, DYF83S1 and DYF51S1, STSs. But in the fertile and RM male groups was found no deletions similar to those, of the infertile males (p=1.0). Instead 4 (13.3%) cases of the RM group males had different micro-deletions included DYS220 (AZFb, sY129), DYS262, DYF8551, and DYF8651, STSs. The AZFc locus of Y-chromosome micro-deletions have a significant role in RM (p=0.045).

**Conclusion:** It seems that the Y-chromosome AZF region's micro-deletions are associated with RM, and we recommend adding this AZF region STSs into infertility analyzing panels.

## Introduction

Recurrent Miscarriage (RM) as one of the common complication of the fertility, is defined as 3 or more consecutive miscarriages. Despite of continuing studies about RM, still the main cause of it is not understood (1). The miscarriage is a misleading word, because it seems that only women are involved in its nature. But it is well recognized that both couples, male or female equally are involved in the occurrence of miscarriage and perhaps in RM (2, 3). 

Recently the changes of male Y-chromosome genetics were introduced as the effective factor in the etiology of RM (3). Micro-deletions of the Y-chromosome with higher incidence have been reported in infertile males than in fertile controls. The Y chromosome 10-Mb AZF region's micro-deletions are one of the most frequently observed structural abnormalities in the male reproductive conditions such as infertility, IVF failures, testicular histology and also recently in the RM (4, 5). The reports revealed a significant association between the "azoospermia factor c" (AZFc) locus and the male infertility. Although fertility may occurs in the presence of partial AZFc deletions with various lengths. The role of these deletions in infertility and miscarriage is controversial. The STSs are known sequences of genomic DNA with less than 1000 bp that can be amplified using a set of primers. It seems that in the AZF region about 300 STSs were mapped, but only a few STSs have been surveyed with infertility (6, 7). 

Several different reports shown that the AZFc region micro-deletion has profound impact on the male fertility and the RM. In the Iranian infertile male population it has been observed that the Y chromosome micro-deletions are common, in addition, an article debated about methodological errors in screening of Yq micro-deletion in Iranian azoospermic males. This article reported that the frequency of AZFb locus micro-deletions are higher than AZFc. 

It seems that the AZFc locus micro-deletions has a great association with Iranian couples with RM (8, 9). The main objective of this article is to survey the incidence of AZFc region's micro-deletion in infertile males with the recurrent miscarriage

## Materials and methods

This is acase-control study that was performed from July 2008 to February 2010. 60 males with a history of infertility complications (infertility and recurrent miscarriage) attending to Yazd Research and Clinical Center for Infertility were selected. The patients depend on their complications were assigned to 2 defined groups. First group; include 30 males with infertility history, and no previous miscarriage events. The second group included 30 male partners of women with at least 3 consecutive miscarriages. Both male and female partners of couples experiencing RPL(second group) were assessed as follows: chromosome analyses were performed on both partners in the couples. 

All women with RPL were checked by ultrasonography and hysterosalpingography for recognition of anatomic abnormalities of the genital tract, had blood drawn for testing hematologic (full blood count, antithrombin III activity, protein C activity, protein S activity, Protrombin 20210 A mutation, factor V Leiden mutation, activated protein C resistance, fasting plasma homocysteine, folic acid and vitamin B_12_) and endocrinologic (fasting glucose, early follicular phase [day 2-5] follicle-stimulating hormone, luteinizing hormone, thyroid-stimulating hormone, prolactin, free testosterone, dehydroepiandrosterone sulfate), immunologic (lupus anticoagulants, anticardiolipin antibodies, antinuclear antibodies, antidouble-stranded DNA) risk factors. The age range and mean age of participants were 22-43 and, 33±6.8 years, respectively.

All male patients’ demographic and clinical data were recorded. The male's complications status predominantly was tested using the standard clinical assessments included sperm count test, hormonal and physical analysis. In addition the control group with 30 healthy men was screened from the same center. The criteria for their selection were no history of miscarriage or other infertility complications, had at least one child and were not oligo or azoospermia.

**Clinical assessments**

In this study 3 technique were utilized; GTG-banding, molecular technique of PCR and sperm counting. Genomic DNA was extracted and purified using the QIAGENE DNA Blood kit (Qiagen, Hilden, Germany). Then DNA was analyzed using simple conventional PCR in 25-mL reactions containing 50 ng of genomic DNA to amplify 8 sequence tagged sites (STS) of AZFc DYF84S1, DYF83S1, DYF51S1, DYF87S, DYS220, DYS262, DYF8551, and DYF8651. After PCR, 2 μl of products were removed from each PCR tube to a new tube, add 10 μl of cresol red/sucrose solution was added to each tube and the products were loaded on a 1% agarose gel visualized in a UV transluminator with ethidium bromide to check that amplification has been successful. 

The 8 AZF region STSs primers that is covering the 3' and 5'interesting flanking region, were designed using the previous study data, the primer perlaquien soft and NCBI website (10). Of all participants a written informed consent was obtained and this study was authorized by the Medical and Biological Research Ethics Committee of Shahid Sadoughi University of Medical Sciences, Yazd.

**Statistical analysis**

The results were evaluated with the Pearson's Χ^2^, Fisher exact test, Student t-test, and the Statistical significance in this study was assumed for p<0.05.

## Results

In this study the number of pregnancy loss was between 3-8. The mean age of male partners of couples with RM, and infertile male groups was 34.7 and 33.5 years, respectively. The demographic data are detailed in table I. Five cases of the infertile group had micro-deletions in one of four AZFC STSs DYF84S1, DYF83S1, DYF51S1, and DYF87S, whereas none of the healthy controls and males of couples with RM groups had micro-deletions in this STSs. Instead, 4 (13.3%) cases of couples with RM had micro-deletions in 1 of 4 different STSs included DYS220, DYS262, DYF8551, and DYF8651 of the AZF region of the Y-chromosome. The results show that the DYS220 STS micro-deletions were more frequent than the other STSs among males of couples with RM. 

In addition, DYF87S STS micro-deletions had a higher frequency than other STSs mutations in this group (Table III). In control males nopreviously described micro-deletions was found. The results of comparing all 3 groups showed that the deletions that caused infertility or miscarriage at AZF region significantly are different (p=0.041). The sperm count test results were reported that, all males of the control group and also male partners of couples with RM had normal sperm count (>20×10^3^), while among infertile males 18 (60%) cases had an abnormal sperm count (36.6% were oligospermia and 23.4% were azoospermia).

**Table I:** Incidence of Y-chromosome micro-deletions for the STSs

**Group**	**DYS220**	**DYS262**	**DYF8551**	**DYF8651**	**DYF84S1**	**DYF83S1**	**DYF51S1**	**DYF87S**	**Case No**
RM	4	3	1	3	-	-	-	-	4
Infertile	-	-	2	-	3	2	2	4	5
Control	-	-	-	-	-	-	-	-	-

RM: Recurrent abortion

## Discussion

The European Academy of Andrology and the European Genetics Quality Network recommend using six STSs to screening the AZF region changes in infertility (11). Y-chromosome micro-deletion is the most frequently observed structural abnormalities in the male specific region of the Y chromosome. Many of men with Y-chromosome micro-deletion have no symptoms but a significant percent of them have fertility complications. 

The studies reported the heterogeneous frequency of the Y chromosome micro-deletion at Middle East infertile male populations, but studies in Iranian infertile males reported a higher frequency than the Middle East (between 5-24.2%) (12). In recent years many studies concentrated on the relation between Y chromosome changes and infertility complications, especially with RM. Previous studies found a significant relation between miscarriage and some mutations of the Y chromosome STSs.

This study results showed that the pathogenic micro-deletions between this groups significantly was different, except DYF8551 STS that had overlapping between infertile male group and males partners of couples with RM. Five cases of infertile males had micro-deletions in the AZFc locus DYF84S1, DYF83S1, DYF51S1, and DYF87S STSs, while none of these alleles observed in the control and RM group males. Of 30 males of RM 4 cases have a micro-deletions in the 4 other AZFc locus STSs including DYS220, DYS262, DYF8551, and DYF8651 (13). 

It is not clearly understood that why different micro-deletions in the AZF region caused different phenotypes. In a study two AZFc, DYF85S1 and DYF86S1, STSs micro-deletions were common in male partner of couples with RM. It seems that the AZF region micro-deletions have a specific consequence of the male fertility. The same authors have reported the different micro-deletions between infertile and RM males (13, 14). 

It seems that AZFc effective microdeletions are different between infertile male and RM male partners. Our findings indicate that the mutations in the RM group had no role in sperm counting problem because in this male group the sperm count was normal, but mutations in the infertile males significantly caused to sperm count reduction. A study that has been reviewed a very wide region of the Y chromosome micro-deletions, have not seen a significant micro-deletion with RM. Currently about 300 known STSs markers were known on the Y chromosome that only a few cases have been surveyed in infertility or RM. 
